# Prevalence and Persistence of Anxiety and Depression over Five Years since Breast Cancer Diagnosis—The NEON-BC Prospective Study

**DOI:** 10.3390/curroncol29030173

**Published:** 2022-03-21

**Authors:** Catarina Lopes, Luisa Lopes-Conceição, Filipa Fontes, Augusto Ferreira, Susana Pereira, Nuno Lunet, Natália Araújo

**Affiliations:** 1 EPIUnit—Instituto de Saúde Pública, Universidade do Porto, Rua das Taipas 135, 4050-600 Porto, Portugal; catarina.lopes@ispup.up.pt (C.L.); luisa.conceicao@ispup.up.pt (L.L.-C.); filipa.fontes@ispup.up.pt (F.F.); susana.pereira@ipoporto.min-saude.pt (S.P.); nlunet@med.up.pt (N.L.); 2 Laboratório para a Investigação Integrativa e Translacional em Saúde Populacional (ITR), Rua das Taipas 135, 4050-600 Porto, Portugal; 3 Instituto Português de Oncologia do Porto, 4200-072 Porto, Portugal; augusto.carmo.ferreira@ipoporto.min-saude.pt; 4 Departamento de Ciências da Saúde Pública e Forenses e Educação Médica, Faculdade de Medicina da Universidade do Porto, 4200-319 Porto, Portugal

**Keywords:** breast cancer, anxiety, depression, longitudinal study

## Abstract

Anxiety and depression are frequent among patients with breast cancer (BCa). Evidence of the persistence and recovery from these conditions and their determinants is scarce. We describe the occurrence of clinically significant anxiety and depression symptoms and their associated factors among BCa patients. A total of 506 women admitted in 2012 at the Portuguese Institute of Oncology of Porto were evaluated before treatment and after one, three, and five years (7.9% attrition rate). The five-year prevalence of anxiety and/or depression (Hospital Anxiety and Depression Scale, subscores ≥ 11) was 55.4%. The peak prevalence for anxiety was before treatment (38.0%), and after one year for depression (13.1%). One in five patients with anxiety/depression at baseline had persistent anxiety/depression over time, while only 11% and 22% recovered permanently from anxiety and depression, respectively, during the first year. Higher education, higher income, practicing physical activity, and adequate fruit and vegetable intake were protective factors against anxiety and/or depression. Loss of job and income, anxiolytics and antidepressants, cancer-related neuropathic pain, and mastectomy were associated with higher odds of anxiety and/or depression. These results highlight the importance of monitoring anxiety/depression during the first five years after cancer diagnosis and identify factors associated with these conditions.

## 1. Introduction

Breast cancer is the most commonly diagnosed cancer, with an estimated 2.3 million new diagnoses worldwide in 2020 [1]. Despite the high incidence of this cancer, the mortality rates have decreased and the relative five-year survival has reached nearly 90% in some countries, which can be explained by improvements in access to screening programs, earlier detection, and progress in therapeutic guidelines and treatment [2].

Patients with breast cancer have been described as being more frequently affected by anxiety and depression than women in the general population, and a wide range of values for the prevalence of anxiety, 20–50%, and of depression, 30–50%, were assessed with questionnaires and reported [3]. This heterogeneity of results may be explained, at least partially, by differences in the instruments used and the timing of assessment after cancer diagnosis [3]. Either during the treatment period and/or post-treatment, the psychological impact can cause difficulties in compliance with therapy, in particular with radiotherapy and chemotherapy, which may contribute to cancer progression and a poorer quality of life [4]. Moreover, anxiety and depression have been associated with breast cancer recurrence and all-cause mortality, and depression with cancer-specific mortality [5]. Additionally, anxiety before treatment was associated with a 50% increased risk of cancer-related neuropathic pain over the first year since breast cancer diagnosis [6], and having clinically significant levels of anxiety and depressive symptoms before treatment and after one year of the diagnosis was associated with cognitive decline over five years in women diagnosed with breast cancer [7]. These results highlight the importance of controlling these psychological disorders.

Most longitudinal studies assessing anxiety and depression had a follow-up of 12 to 18 months, and few reported on the change in anxiety and depression status over time [8]. A recent study with longer follow-up reported a shift towards worse anxiety and depression status (normal, mild, moderate, and severe anxiety/depression) at five to six years than at 40 weeks after cancer diagnosis in about one-third of the sample [9]. Accordingly, more research studies with longer follow-up are needed to understand the persistence and recovery of these psychological symptoms. Moreover, studies reporting on the determinants of these conditions are scarce [3]. Results from cross-sectional studies suggest that social isolation, unmet needs, and social support may influence the levels of anxiety and depression among women with breast cancer [10,11].

Therefore, this study aims to describe the occurrence of clinically significant anxiety and depressive symptoms among women with breast cancer over a five-year follow-up since cancer diagnosis. We focus on the evolution of these psychological symptoms over time, namely, the proportions of persistent, recovery and incident cases, and, as a secondary objective, we present the associations between socio-demographic, lifestyle, and clinical characteristics with these psychological conditions. 

## 2. Materials and Methods

### 2.1. The NEON-BC Cohort

The present study is based on the prospective study NEON-BC, which aimed to investigate the neurological complications of breast cancer and its treatments. The initial cohort included women consecutively recruited in 2012 among those with newly diagnosed breast cancer proposed for surgery at the Breast Clinic of the Portuguese Institute of Oncology of Porto, Portugal. The study methodology has been described in detail elsewhere [12]. Briefly, women who did not have a history of chemotherapy or radiotherapy treatment for another primary cancer, had no previous breast surgery, and were able to understand the purpose of the study were invited to participate. Those who presented a score lower than 17 (or 16, if they were aged 65 years or more) at the cognitive screening test Montreal Cognitive Assessment (MoCA), were excluded due to the high probability of cognitive impairment [13] and low ability to answer to self-questionnaires.

**Figure 1:**
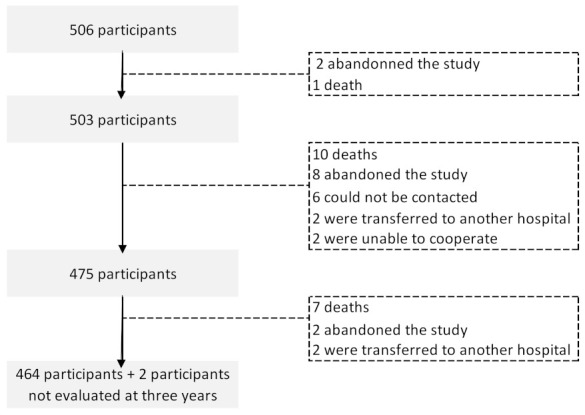
Flowchart describing the number of breast cancer patients in each of the follow-up evaluations.

A total of 506 participants were assessed at baseline, before any cancer treatment; 503, 475, and 466 were evaluated at one, three, and five years after diagnosis, respectively. Among those lost to follow-up, 18 died, 12 abandoned the study, four were transferred to another hospital, two were unable to participate, and four could not be contacted (Figure 1). Two participants were evaluated at the five-year evaluation but not at three years. Participants lost to follow up were older (mean, sd: 58.2, 15.1 vs. 54.4, 10.7; *p* = 0.028) but had similar levels of education (mean, sd: 6.8, 4.2 vs. 7.7, 4.5; *p* = 0.154), and anxiety (mean, sd: 4.5, 8.7, 4.7 vs. 9.3, 4.2; *p* = 0.340) and depression scores at baseline (mean, sd: 5.3, 4.3 vs. 5.3, 3.6; *p* = 0.988). 

### 2.2. Measures

Sociodemographic characteristics and lifestyles were assessed through interviews using a structured questionnaire. Date of birth, number of complete years of education, marital status (single, married, living with partner, divorced, widow), employment (full-time and part-time employment, unemployed, retired, sick leave, housewife, student), name of regular medication, and previous diagnosis of hypertension and of diabetes were self-reported by participants at baseline and at the three- and five-year evaluations. The category of individual monthly income (500€ or lower, 501–1000€, 1001–1500€, 1500–2000€, and >2000€) and lifestyles (frequency of consumption of one typical alcoholic beverage, self-classification as current, ex-smoker and never smoker, number of fruit and vegetables pieces consumed per day, and self-classification of practicing or not physical activity) was obtained at the three- and five-year evaluations; baseline levels of exposure were assessed retrospectively, at the three-year assessment. Loss of income was considered when a change to an inferior category of income was reported.

Clinical characteristics, namely, breast cancer stage classified according to the *American Joint Committee on Cancer Tumor, Node, Metastases classification* 7th edition [14], and treatment details were abstracted from clinical files at baseline, and after one, three, and five years of follow up. Breast cancer subtype was attributed based on information from medical files regarding immunohistochemistry and in situ-hybridization-based biomarkers, namely, hormone receptors (HR; estrogen and progesterone receptors, considered positive if present in ≥1% of cells) and human epidermal growth factor receptor (HER2), and was classified into HR-positive/HER2-negative (HR+/HER2−); HER2-positive (HER2+); triple-negative breast cancer (HR-negative/HER2-negative).

The Hospital Anxiety and Depression Scale (HADS) [15] was used to assess anxiety and depression symptoms. It is a multiple-choice questionnaire with 14 questions, seven regarding depression (HADS-D subscale) and seven regarding anxiety (HADS-A subscale). Each item is scored from 0 to 3 and the maximum score for each subscale is 21. The validation study of the Portuguese version of the HADS showed psychometric properties similar to those in international studies, namely, the existence of two factors—anxiety and depression—adequate reliability for the anxiety and depression subscales (α Cronbach of 0.76 and 0.81, respectively), 15-days test-retest correlations of 0.75 for anxiety scores and 0.75 for depression scores, and adequate content validity, suggesting that it measures the same constructs, in the same way, as the original HADS form [16]. The HADS is suitable for screening anxiety and depression, as demonstrated in the following meta-analysis: the pooled sensitivity and specificity of the HADS subscales to detect diagnosed cases of anxiety were 48.7%, 78.7%, and 71.6%, 82.6%, to detect depression, using the original classification of the author [17]. 

According to this classification, we defined clinically significant anxiety symptoms and clinically significant depressive symptoms as a score in respective subscale equal or higher than 11; scoring between 8 and 10 was considered a borderline disorder, and those scoring below 8 were considered as ‘normal’.

Changes in anxiety/depression status over time were defined as follows: at each evaluation, participants were classified as having persistent anxiety/depression if their HADS-A/HADS-D scores were consistently equal to or higher than 11 in all previous and present evaluations; permanent recovery from anxiety/depression corresponds to consistent HADS-A/HADS-D scores equal to or higher than 11 in all previous evaluations and lower than 8 in the present and next evaluations (e.g., cases of recovery from anxiety at the three-year assessment are participants who had anxiety at baseline and at one-year and HADS-A score < 8 at the three- and five-year evaluations).

### 2.3. Statistical Analysis

Patients’ characteristics were presented as counts and proportions for all categorical variables. Five-year period and point prevalence estimates at each evaluation with corresponding 95% confidence intervals (95%CI) were estimated for anxiety and depression. The McNemar’s test was used to compare the proportion of patients with each psychiatric complication at baseline, one, three, and five years. 

Adjusted odds ratios (aOR) and 95%CI were computed using logistic regression to quantify the relation between sociodemographic, lifestyles and clinical variables, and the occurrence of anxiety and depression at least once over the follow-up period, considering the entire cohort (prevalent cases of clinically significant anxiety/depression symptoms) or excluding participants with clinically significant anxiety or depressive symptoms at baseline (incident cases of clinically significant anxiety/depression symptoms).

For the main objective of describing the proportions of persistent cases, of women who recovered from anxiety/depression, and of incident cases at each evaluation, a sample of 319 allows estimation of proportions up to 30% with a 95% confidence interval with a margin of error of 4%. For the secondary objective of identifying associations between patient’s and tumor-related characteristics with anxiety/depression, the available sample size of the cohort allows to detect OR ≥ 3, considering a significance level of 5%, a power of 80%, a prevalence of the exposition (patient’s or tumor-related characteristics) of at least 20% (ratio non-exposed:exposed = 4) and a prevalence of the outcome (persistent cases, recovery, incident cases) of 10% in the non-exposed or reference category.

Statistical analyses were conducted using STATA^®^, version 15.1 (StataCorp, College Station, TX, USA).

### 2.4. Ethic Approval

The study was conducted according to the guidelines of the Declaration of Helsinki and approved by the Ethics Committee of the Portuguese Institute of Oncology of Porto (ref. CES 406/011, CES 99/014 and CES 290/014) and the National Data Protection Authority (ref. 9469/2012 and 8601/2014). All participants gave their written informed consent.

## 3. Results

### 3.1. Participants Sociodemographic, Lifestyles and Clinical Characterization

Participants’ sociodemographic, lifestyle and clinical characteristics are presented in Table 1. At baseline, among the 506 participants, 50.2% were younger than 55 years of age, 71.4% had up to nine years of education, and 55.5% were professionally active (holding part-time or full-time employment). A total of 33.4% and 9.9% reported hypertension and diabetes, respectively. Only 3.6% had had a previous cancer diagnosis, and the majority reported consuming chronic medicines (64.2%); 19.4% reported using anxiolytics and 18.8% reported using antidepressants. A total of 53.5% of all of the women were diagnosed with stage 0 (ductal in situ carcinoma) or I, and the most frequent type of cancer was HR+/HER2− (76.4%), followed by HER2+ (15.4%) and triple negative (8.2%).

During the first year after diagnosis, all participants underwent breast surgery except one who had axillary surgery only and 97.0% also received neoadjuvant or adjuvant treatments, namely, chemotherapy (59.2%), hormone therapy (83.9%), and radiotherapy (73.6%). During the five-year follow-up, 16 women had breast cancer recurrence and 25 had a new primary tumor (Table 2).

### 3.2. Prevalence of Anxiety and Depression Overt the 5-Year Follow-Up

At baseline (before treatment), 38.0% of the participants had clinically significant levels of anxiety. These values decreased at the one-year evaluation (25.3%, *p* < 0.001) and even further at the three-year assessment (21.1%, *p* = 0.031), with a slight increase at the five-year assessment (25.4%; *p* = 0.066). Among women who did not have anxiety prior to treatment (62.0%), 12.1% developed anxiety in the first year, 7.0% in the third year, and 4.2% in the fifth year. A total of 18.2% of the participants with anxiety at baseline presented anxiety in all evaluations, and a few had permanently recovered from anxiety. Most participants had fluctuating anxiety status over the five years of follow-up. The prevalence of depression was 8.1% at baseline and increased significantly to its highest value, 13.1%, at the first year of follow-up (*p* = 0.004). At five years, 10.3% reported clinical depressive symptomatology and 17.0% of those who had depression at baseline had normal HADS-D scores (Table 3).

The five-year period prevalence of having clinically significant symptoms of anxiety was 55.4% [95%CI: 50.9, 60.0], being the value of 25.5% [95%CI: 21.6, 29.7] for depression, and 55.5% [95%CI: 51.1–59.9] for having clinically significant symptoms of either anxiety or depression.

### 3.3. Factors Associated with Anxiety and Depression

**Figure 2:**
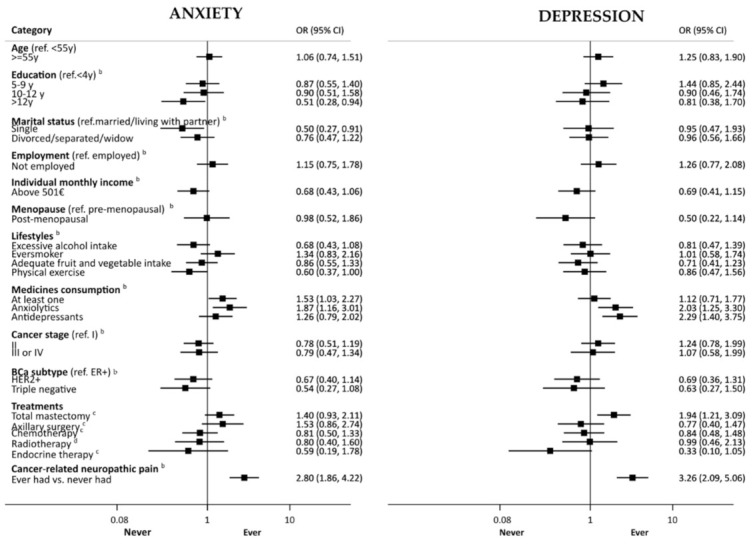
Association of baseline factors with having had clinically significant anxiety/depression symptoms (anxiety/depression sub score of the Hospital Anxiety and Depression Scale equal or higher than 11) at any evaluation during the five-year follow-up (prevalent cases). BCa, breast cancer; LND, lymph node dissection. Black boxes and horizontal lines represent OR and corresponding 95% CI, respectively, according to the log scale in the horizontal axis. ^a^—adjusted for age; ^b^—adjusted for age and education; ^c^—adjusted for age, education, cancer stage and subtype; ^d^—adjusted for age, education, cancer stage and subtype, breast surgery.

Figure 2 depicts the association between baseline factors, such as sociodemographic, lifestyle and clinical characteristics of participants, and having had clinically significant anxiety or depression symptoms at any evaluation during the five-year follow-up (prevalent cases). Participants with more than 12 years of education had lower odds of having clinical levels of anxiety (age-adjusted OR = 0.51, 95%CI: 0.28, 0.94). Being single was associated with lower odds of anxiety compared to being married/living with a partner (adjusted OR [aOR] = 0.50, 95%CI: 0.27, 0.91). Participants reporting practicing physical activity at baseline were less likely to have anxiety during follow-up (aOR = 0.60, 95%CI: 0.37, 1.00). Patients who reported the regular consumption of at least one medicine were more likely to have clinically significant symptoms of anxiety (aOR = 1.53, 95%CI: 1.03, 2.27). The consumption of anxiolytics at baseline was positively associated with having clinically significant anxiety and depressive symptoms over the follow-up period (aOR = 2.80, 95%CI: 1.86, 4.22 and, aOR = 2.03, 95%CI: 1.25, respectively), and patients who used antidepressants experienced more frequent clinically significant depressive symptoms (aOR = 2.29, 95%CI: 1.40, 3.75).

Mastectomy vs. breast conserving surgery was associated with higher odds of clinically significant depressive symptoms (aOR = 1.94, 95%CI: 1.21, 3.09). Patients with cancer-related neuropathic pain presented more often with clinically significant anxiety and depressive symptoms (aOR = 2.80, 95%CI: 1.86, 4.22, and aOR = 3.26, 95%CI: 2.09, 5.06, respectively). Participants with monthly income above 500€, post-menopausal, or treated with endocrine therapy tended to be less likely to present clinically significant depressive symptoms, although estimates did not reach significance. 

Additionally, associations of baseline factors, changes in socio-demographic and clinical characteristics, and treatments with having clinically significant anxiety/depression symptoms at any evaluation during the five-year follow-up, among participants without clinically significant levels of anxiety/depression at baseline (incident cases) are presented in Table 4. Having breast cancer stage II vs. 0/I was associated with lower odds of clinically significant anxiety (aOR = 0.43, 95%CI: 0.22, 0.85), as well as having a triple negative breast cancer subtype compared to the HR+/HER2− subtype (aOR = 0.12, 95%CI: 0.02, 0.95), and having cancer-related neuropathic pain was associated with higher odds of anxiety and depression (aOR = 2.43, 95%CI: 1.35, 4.36, and aOR = 2.72, 95%CI: 1.63, 4.55; respectively). Loss of job and loss of income were associated with higher odds of depression (aOR = 2.62, 95%CI: 1.10, 6.20 and aOR = 1.99, 95%CI: 1.16, 3.41, respectively), while participants who reported a consumption of at least five pieces of fruit and vegetables per day at baseline were less likely to have depression (aOR = 0.47, 95%CI: 0.22, 0.99). The consumption of antidepressants was positively associated with clinically significant depressive symptoms (aOR = 2.06, 95%CI: 1.16, 3.68). Participants who underwent total mastectomy were more likely to present clinically significant depressive symptoms than patients who had breast conserving surgery (aOR =1.87, 95%CI: 1.08, 3.23).

## 4. Discussion

More than half of the patients with breast cancer had anxiety or depression at least once during the five years of follow-up, mostly due to the prevalence of anxiety, which was the highest before treatment (38%), while the prevalence of depression was the highest after treatment, especially one year after (13%). Higher education, higher income, practice of physical activity, and adequate fruit and vegetable intake were protective factors in relation to anxiety and/or depression, while loss of job, lower income, use of anxiolytics and antidepressants, cancer-related neuropathic pain, and mastectomy were associated with higher odds of clinically significant anxiety and/or depressive symptoms.

A previous study reported the following changes in depression clinically diagnosed over the first year after breast surgery: among the 82 participants with depression at baseline, 31.0% had persistent depression and 69.0% had recovered from this condition; the incidence of depression was 13.0% [18]. The persistence of depression and the incidence at one year could be considered similar to the findings of the present study, taking into account the low number of incident and persistent cases in both studies and the precision of the estimates. Our results are lower regarding recovery (22.0%), although we reported permanent recovery considering the next two assessments at three and five years. Indeed, in the NEON-BC cohort, we observed a higher result if we consider only the baseline and one-year assessments as follows: 46.3% of patients with clinically significant depression at baseline had scores within the normal range at one year, but part of these women had a recurrence of depressive symptoms after the one-year evaluation. The longer follow-up assessed in the NEON-BC cohort and the lower value for permanent recovery than for recovery at one year highlight the importance of assessing depression not only during the first year of treatment but also afterwards.

In the NEON-BC, one-quarter of participants had clinically significant levels of anxiety at the five-year evaluation, whereas for depression the value was lower (10.3%). These results are similar to previous findings of 26.3% and 9.8% [19].

As reported in previous studies, higher levels of education are associated with fewer anxious and depressive symptomatology and in women dealing with breast cancer, having lower levels of education may increase the probability of having one of the two psychological disorders (four times more likely) [20,21,22]. We found an inverse association between education level higher than 12 years and anxiety, which could be explained by the higher access and comprehension of health-related information regarding the condition itself, the treatment plan, and its possible consequences, reducing the uncertainty significantly [21].

In our study, being married or living with a partner was associated with anxiety. In a study with 653 women with breast cancer, the authors found that married individuals entail a higher psychological burden than not being married (single, widow, or divorced) [23]. However, other studies stated that there were fewer symptoms of anxiety and depression among women who were married or living with a partner, since they perceived more social and financial support [21,24]. Therefore, further research is needed regarding this topic.

Financial distress is another risk factor for having clinical depression and anxiety. Studies regarding this specific topic are not consensual. On one hand, studies point towards a lower probability of returning to work three years post-breast cancer diagnosis due to previous depressive symptoms [25], but on the other hand, there is a lack of research with long term follow-up that highlights this mutual significant impact [26]. In the present study, only job loss was associated with developing depression, and low income at baseline was marginally associated with anxiety and depression at any evaluation (at baseline or during follow-up).

Practicing physical activity and consuming the amount of five pieces of fruit and vegetables per day were found to be protective factors against anxiety and depression, respectively. Other studies have attested that in breast cancer patients, exercise and a balanced diet can improve health-related quality of life as well as lower the depressive effects of cancer itself and its treatments [27,28].

Women who submitted to total mastectomy experienced depression more frequently than those who had breast conserving surgery, a result that is in accordance with a recent meta-analysis [29]. Mastectomy is associated with loss of femininity, dissatisfaction with body image, and lower self-esteem that may contribute to changes in sexual life and social interactions [30], and ultimately to depressive symptomatology.

Lastly, having cancer-related neuropathic pain was significantly associated with anxiety and depression in our study. Other studies have reported similar results, namely Schou Bredal et al. [31], who compared breast cancer patients with neuropathic pain to breast cancer patients without neuropathic pain and found significant differences in the levels of anxiety and depression between both groups, supporting the association between these psychological variables with neuropathic pain. However, it remains unclear which occurred first. In the present study, among participants with no clinically significant symptoms of depression at one year, those with depression at baseline were more likely to have cancer-related neuropathic pain at one year (data not shown), suggesting that depressive symptoms precede neuropathic pain.

### Strengths and Limitations

The NEON-BC cohort had a high retention rate (92.1% of participants at baseline were evaluated at five years), which contributed to the validity of the results. The sample size, selection criteria, and assessment of multiple variables of sociodemographic, lifestyle, medication, and clinical information contributed to identifying the determinants of anxiety and depression, although the small number of persistent and permanently recovered cases of anxiety and depression precluded the study of the determinants of these conditions. Moreover, we used a self-reported measure, the HADS, to assess anxiety and depression levels, and problems in the interpretation of certain items may have reduced the internal validity of our results.

Nevertheless, our results reflect the experience of women with early-stage breast cancer, as they represented almost all of the NEON-BC cohort. Moreover, the external validity of our study is limited because only one hospital was involved. However, the Portuguese Institute of Oncology of Porto represents the largest hospital providing cancer care in northern Portugal and is the reference hospital for a large geographical area.

## 5. Conclusions

Clinically significant anxiety and depressive symptoms affected nearly half the women with breast cancer over the first five years after cancer diagnosis, and anxiety and depression persisted in one fifth of the affected women before treatment. These results emphasize the need for clinical and psychological support and intervention not only at diagnosis and treatment, but also in the long-term. Patients’ socio-demographic characteristics and lifestyles, having undergone mastectomy, and suffering from neuropathic pain were identified as factors that could be considered for the design of future interventions that would aim to reduce anxiety and depression among breast cancer survivors.

## Figures and Tables

**Table 1:** Baseline socio-demographics characteristics of the participants, lifestyles, and comorbidities, as well as clinical characteristics of the breast tumor.

Variable (Number of Respondents)			*n* (%)
**Socio-demographics**			
	Age (years) (*n* = 506)		
		<55	254 (50.2)
		≥55	252 (49.8)
	Education (years) (*n* = 506)		
		≤4	216 (42.7)
		5–9	145 (28.7)
		≥10	145 (28.7)
	Living in Greater Porto area (*n* = 506)		226 (44.7)
	Marital status (*n* = 506)		
		Married/living together	353 (69.8)
		Single	53 (10.5)
		Widower/divorced	100 (19.8)
	Employed (*n* = 503)		279 (55.5)
	Individual monthly income above 500€ (*n* = 467)		210 (45.0)
	Post-menopausal (*n* = 506)		288 (56.9)
**Lifestyles**			
	Alcohol consumption, more than 10 g/day (*n* = 505)		97 (19.2)
	Ever smoker (*n* = 475)		97 (20.4)
	Fruits and vegetables ≥ 5 portions/day (*n* = 472)		104 (22.0)
	Practicing physical activity (*n* = 475)		80 (16.8)
**Comorbidities**			
	Hypertension (*n* = 506)		169 (33.4)
	Diabetes (*n* = 506)		50 (9.9)
	Previous cancer (*n* = 475)		17 (3.6)
	Chronic medicines consumption (*n* = 462)		
		None	181 (35.8)
		One	85 (16.8)
		Two to five	161 (31.8)
		More than five	79 (15.6)
		Anxiolytics	98 (19.4)
		Antidepressants	95 (18.8)
**Clinical characteristics of the breast tumor**			
	Cancer stage (*n* = 505)		
		0/I	270 (53.5)
		II	156 (30.9)
		III/IV	79 (15.6)
	Breast cancer subtype (*n* = 474)		
		HR+/HER2−	363 (76.4)
		HER2+	73 (15.4)
		Triple negative	39 (8.2)

HER2+, human epidermal growth factor receptor positive; HR+/HER2−, estrogen and progesterone receptors positive and human epidermal growth factor receptor (HER2) negative.

**Table 2:** Breast cancer treatments and changes in clinical and socio-demographic characteristics of the participants, over the five-year follow-up.

	Period of Follow-Up		
	During the First Year	Between the First and the Third Year	Between the Third and the Fifth Year
	*n* (%)	*n* (%)	*n* (%)
**Breast cancer treatment**			
Breast conserving surgery ^a^	250 (49.5)	0	0
Mastectomy ^a^	254 (50.3)	0	0
Breast reconstruction ^a^	58 (11.5)	26 (5.5)	34 (7.3)
Axillary lymph node dissection ^a^	174 (34.5)	1 (0.2)	0
Chemotherapy ^b^	298 (59.2)	8 (1.7)	9 (1.9)
Radiotherapy ^b^	370 (73.6)	6 (1.3)	2 (0.4)
Endocrine therapy ^b^	422 (83.9)	394 (82.9)	381 (81.8)
Targeted therapy ^b^	67 (13.3)	3 (0.6)	3 (0.6)
**Breast cancer recurrence ^b^**	6 (1.2)	7 (1.5)	3 (0.7)
**New primary tumor ^b^**	4 (0.8)	11 (2.2)	10 (2.1)
**Changes in socio-demographic conditions**			
Became widowed/divorced/separated ^c^	13 (3.7)	17 (5.0)	9 (3.0)
Loss of job ^d^	not evaluated	24 (9.8)	5 (2.1)
Became retired ^d^	not evaluated	27 (11.1)	14 (5.7)
Loss of income ^e^	not evaluated	48 (10.3)	98 (20.5)

^a^ Data presented for the first year of follow-up were obtained from 505 women 15 days after breast surgery; for the period between the one- and the three-year evaluations and the three- and the five-year evaluations, data were collected from 475 and 466 participants, respectively. ^b^ Data presented for the first year of follow-up were obtained from 503 women at the one-year evaluation; for the period between the one- and the three-year evaluations and the three- and the five-year evaluations data were collected from 475 and 466 participants, respectively. ^c^ Data presented refer to the following women who were married or living with a partner at baseline and responded at the one-, three-, and five-year evaluations: 353, 338, and 304 women, respectively. ^d^ Data refer to women who were employed at baseline and responded at the three- (*n* = 244) and five-year (*n* = 244) evaluations. ^e^ Data refer to 467 and 463 women who responded at the three- and five-year evaluations.

**Table 3:** Occurrence, persistence, and recovery from clinically significant anxiety and depressive symptoms during the five-year follow-up, assessed with the Hospital Anxiety and Depression Scale (HADS).

	Baseline	One-Year	Three-Year	Five-Year
	*n* (%)	*n* (%)	*n* (%)	*n* (%)
**Anxiety**				
Prevalent cases	192 (38.0)	127 (25.3)	100 (21.1)	118 (25.4)
Incident cases ^a^	-	38 (12.1)	22 (7.0)	13 (4.2)
Permanent recovery ^b^	-	21 (10.9)	10 (5.2)	3 (1.6)
Persistent anxiety ^c^	-	89 (46.4)	46 (24.0)	35 (18.2)
**Depression**				
Prevalent cases	41 (8.1)	66 (13.1)	43 (9.1)	48 (10.3)
Incident cases ^a^	-	50 (10.8)	16 (3.4)	13 (2.8)
Permanent recovery ^b^	-	9 (22.0)	0	7 (17.0)
Persistent depression ^c^	-	16 (39.0)	8 (19.5)	7 (17.0)

Participants who completed the anxiety and depression sub-scales were: 505 and 506, respectively, at the one-year evaluation; 503 and 503, at the one-year evaluation; 475 and 474, at three-years; 464 and 466 at the five-year evaluation. ^a^ Participants who had HADS-A/HADS-D below 11 in all previous evaluations and HADS-A/HADS-D equal to or higher than 11 in the present evaluation. ^b^ Participants who had an anxiety/depression score in the Hospital Anxiety and Depression Scale (HADS-A/HADS-D) equal to or higher than 11 in all previous evaluations and then present a HADS-A/HADS-D score lower than 8 at the present and future evaluations. ^c^ Participants who had HADS-A/HADS-D equal to or higher than 11 in all previous and present evaluations.

**Table 4:** Association of baseline factors, changes in socio-demographic and clinical characteristics, and treatments with having clinically significant anxiety/depression symptoms (anxiety/depression sub score of the Hospital Anxiety and Depression Scale [HADS-A/HADS-D] equal or higher than 11) at any evaluation during the five-year follow-up, among participants with HADS-A/HADS-D < 11 at baseline (incident cases).

	Anxiety during Follow-Up (Ever vs. Never)	Depression during Follow-Up (Ever vs. Never)	
	OR [95% CI]	OR [95% CI]	
Age (years) ≥55 vs. <55	1.20 [0.71, 2.05]	1.09 [0.67, 1.77]	
Education (years) 5–9 vs. ≤4	1.08 [0.55, 2.12]	1.36 [0.74, 2.51]	a
Education (years) 10–12 vs. ≤4	1.46 [0.67, 3.17]	0.78 [0.35, 1.74]	b
Education (years) >12 vs. ≤4	0.33 [0.10, 1.05]	0.64 [0.25, 1.60]	b
Post-menopausal vs. pre-menopausal	1.02 [0.38, 2.68]	0.57 [0.22, 1.44]	b
Single vs. married/living with partner	0.35 [0.12, 1.07]	1.11 [0.50, 2.46]	b
Widow/divorced/separated vs. married/living with partner	0.70 [0.34, 1.42]	0.92 [0.47, 1.77]	b
Became widowed/divorced/separated	2.10 [0.79, 5.57]	1.72 [0.76, 3.89]	b
Employed vs. unemployed	0.93 [0.49, 1.75]	1.55 [0.87, 2.77]	b
Lost job vs. still employed	1.62 [0.52, 5.08]	2.62 [1.10, 6.20]	b
Retired vs. still employed	0.59 [0.21, 1.65]	1.52 [0.70, 3.33]	b
Individual monthly income ≤500€ vs. >500€	0.51 [0.26, 1.01]	0.76 [0.42, 1.38]	b
Lost income	1.16 [0.61, 2.23]	1.99 [1.16, 3.41]	b
Alcohol >10 g/day vs. ≤10 g/day	0.73 [0.36, 1.46]	0.84 [0.45, 1.58]	b
Ever smoker vs. never smoker	1.29 [0.65, 2.58]	1.22 [0.65, 2.27]	b
Fruit and vegetables ≥5 pieces/day vs. <5/day	0.68 [0.34, 1.38]	0.47 [0.22, 0.99]	b
Practicing physical activity vs. not	0.51 [0.22, 1.17]	0.67 [0.31, 1.45]	b
Consumption of at least one medicine vs. none	1.13 [0.63, 2.02]	1.06 [0.62, 1.81]	b
Anxiolytics vs. no anxiolytics	1.46 [0.73, 2.91]	1.54 [0.85, 2.78]	b
Antidepressants vs. no antidepressants	1.36 [0.70, 2.63]	2.06 [1.16, 3.68]	b
Cancer stage II vs. 0/I	0.43 [0.22, 0.85]	1.27 [0.73, 2.20]	b
Cancer stage III/IV vs. 0/I	0.61 [0.27, 1.35]	0.96 [0.46, 2.03]	b
Subtype HER2+ vs. HR+/HER2−	0.87 [0.41, 1.82]	0.88 [0.44, 1.76]	b
Subtype Triple negative vs. HR+/HER2−	0.12 [0.02, 0.95]	0.39 [0.11, 1.32]	b
Mastectomy vs. breast conserving surgery	1.25 [0.67, 2.36]	1.87 [1.08, 3.23]	c
Breast reconstruction vs. none	0.95 [0.44, 2.05]	0.82 [0.42, 1.59]	d
Axillary surgery vs. LND	1.01 [0.40, 2.55]	0.65 [0.30, 1.38]	c
Chemotherapy vs. no chemotherapy	0.60 [0.29, 1.26]	0.84 [0.43, 1.63]	c
Radiotherapy vs. no radiotherapy	0.47 [0.15, 1.51]	0.77 [0.31, 1.94]	e
Endocrine therapy vs. no endocrine therapy	0.58 [0.12, 2.80]	0.31 [0.09, 1.06]	c
Trastuzumab vs. no trastuzumab	0.17 [0.01, 2.22]	0.57 [0.05, 6.19]	c
Cancer-related neuropathic pain anytime during the Follow-up vs. never	2.43 [1.35, 4.36]	2.72 [1.63, 4.55]	b
Recurrence	0.83 [0.09, 7.85]	0.68 [0.08, 5.72]	b
Second primary cancer	1.33 [0.39, 4.57]	0.75 [0.21, 2.63]	b

BCa, breast cancer; HER2+, human epidermal growth factor receptor positive; HR+/HER2−, estrogen and progesterone receptors positive and human epidermal growth factor receptor (HER2) negative; LND, lymph node dissection. a—adjusted for age; b—adjusted for age and education; c—adjusted for age, education, cancer stage and subtype; d—adjusted for age, education, cancer stage and subtype, breast surgery; e—adjusted for age, education, cancer stage and subtype, breast surgery and axillary surgery.

## Data Availability

The datasets generated and analyzed in this study will not be publicly available given that the included patients do not specifically provide their consent for the public sharing of their data, and that anonymization is unlikely to be feasible, since the identification of patients treated in only one institution within a relatively short period may be possible when taking the socio-demographic and clinical characteristics into account.
